# The genetic variant *SLC2A1*-rs1105297 is associated with the differential analgesic response to a glucose-based treatment in newborns

**DOI:** 10.1097/j.pain.0000000000003051

**Published:** 2023-09-13

**Authors:** Riccardo Farinella, Fabio Falchi, Arianna Tavanti, Cristina Tuoni, Maria Grazia Di Nino, Luca Filippi, Massimiliano Ciantelli, Cosmeri Rizzato, Daniele Campa

**Affiliations:** a Department of Biology, University of Pisa, Pisa, Italy; b Division of Neonatology, Santa Chiara Hospital, Pisa, Italy; c Neonatology and Neonatal Intensive Care Unit, Department of Clinical and Experimental Medicine, University of Pisa, Pisa, Italy; d Centro Di Formazione e Simulazione Neonatale “NINA”, Azienda Ospedaliero-Universitaria Pisana, Pisa, Italy; e Department of Translational Research and of New Technologies in Medicine and Surgery, University of Pisa, Pisa, Italy

**Keywords:** genetics, genetic variability, neonates, newborn, pharmacogenetics, precision medicine, personalized medicine, glucose, glucose transporters, SLC2A1, SLC2A2, noradrenaline, noradrenaline transporter, SLC6A2

## Abstract

Neonatal pain is a critical issue in clinical practice. The oral administration of glucose-based solutions is currently one of the most common and effective nonpharmacologic strategies for neonatal pain relief in daily minor procedures. However, a varying degree of analgesic efficacy has been reported for this treatment. Environmental, maternal, and genetic factors may explain this variability and potentially allow for a personalized analgesic approach, maximizing therapeutic efficacy and preventing side effects. We investigated the exposome (ie, the set of clinical and anthropometric variables potentially affecting the response to the therapy) and the genetic variability of the noradrenaline transporter gene (solute carrier family 6 member 2 [*SLC6A2*]) and 2 glucose transporter genes (solute carrier family 2 member 1 [*SLC2A1*] and 2 [*SLC2A2*]) in relation to the neonatal analgesic efficacy of a 33% glucose solution. The study population consisted in a homogeneous sample of more than 1400 healthy term newborns. No association for the exposome was observed, whereas a statistically significant association between the G allele of *SLC2A1*-rs1105297 and a fourfold decreased probability of responding to the therapy was identified after multiple-testing correction (odds ratio of 3.98, 95% confidence interval 1.95-9.17; *P* = 4.05 × 10^−4^). This allele decreases the expression of *SLC2A1-AS1*, causing the upregulation of *SLC2A1* in the dorsal striatum, which has been suggested to be involved in reward-related processes through the binding of opioids to the striatal mu-opioid receptors. Altogether, these results suggest the involvement of *SLC2A1* in the analgesic process and highlight the importance of host genetics for defining personalized analgesic treatments.

## Abstract 2

Supplemental Digital Content is Available in the Text.

The genetic variability of *SLC2A1* gene affects the neonatal response to a 33% glucose-based analgesic treatment, highlighting the importance of host genetics for personalized analgesia.

## 1. Introduction

Pain is a medical issue in neonatal clinical practice, and several studies identified an association between neonatal pain and pain-related stress with short and long-term adverse health outcomes, such as sleep disorders,^1^ reduced neuroanatomical development,^27^ alterations in pain sensitivity,^37^ and neurological disorders,^3,16^ which may last until infancy and childhood.^2^

Specific analgesic strategies have been developed to prevent neonatal pain.^33^ Pharmacological approaches based on opioids, benzodiazepines, or other analgesic drugs are recommended for the most painful and invasive procedures, whereas nonpharmacological strategies are indicated for daily treatments such as heel prick and venipuncture.^28,33,39^ One of the most common and effective nonpharmacological treatment is the oral administration of a 20% to 33% glucose solution.^10^ Despite the high analgesic efficacy of this therapy, a fraction of newborns does not respond to the treatment, suggesting the involvement of host genetic factors.

Pharmacogenetic studies have already demonstrated the relevance of the genetic variability in determining interindividual differences in the response to specific analgesic treatments.^34,36^ For example, polymorphisms in *OPRM1, COMT*, and *CYP2D6* genes have been associated with the efficacy of opioids-based analgesia.^9,11,47^ Some of these associations have been translated into clinical practice accounting for the genetic variability of the patient, when prescribing a pharmacological treatment.^9,13^ For example, the Food and Drug Administration suggests testing for *CYP2D6* genotypes for codeine, oliceridine, and tramadol treatments and for *CYP2C9* genotypes for meloxicam treatment.^21,43^

The identification of genetic variants influencing the response to a treatment, in addition to allowing for the potential personalization of the therapy, can provide evidence for the mechanism of action of a drug.^18,25,49^ The mechanism by which glucose induces neonatal analgesia is still unclear, but one of the main hypotheses proposes the involvement of the endogenous opioid system.^10,19,33^ Accordingly, we reported in a previous study the association between a polymorphic variant, the missense mutation rs1799971, which belongs to the mu-opioid receptor (*OPRM1*) gene, and a reduced analgesia in 1077 healthy term newborns treated with oral glucose.^17^

Response to the treatment is a complex trait, and it is likely that many genetic polymorphisms may affect its therapeutic efficacy. With these premises, the goal of this study was to use genetic variability to identify in advance subjects nonresponding to the glucose-based treatment to allow for a different and personalized approach.

Many studies suggested a role for the norepinephrine transporter (NET) in opioids-mediated analgesia, highlighting a relationship between opioids and the noradrenergic system.^7,42,45^ We investigated whether the genetic variability of the solute carrier family 6 member 2 (*SLC6A2*) gene, coding for NET, could influence the response to the analgesic treatment based on glucose administration.

Moreover, since glucose is the effector of the neonatal analgesia, we hypothesize that genetic variants involved in glucose transport and metabolism may play a role in the response to the therapy. Thus, we also investigated whether single nucleotide polymorphisms (SNPs) within the solute carrier family 2 member 1 and 2 (*SLC2A1* and *SLC2A2*) genes, coding for the ubiquitous glucose transporter GLUT-1 and the low-affinity glucose transporter GLUT-2, respectively, could affect the response to the therapy since SNPs in these 2 genes have been associated with hematic glucose concentration through genome-wide association studies.^12,23^

## 2. Materials and methods

### 2.1. Study subjects

The study was performed on 1421 healthy full-term newborns, enrolled between 2015 and 2022 at the Neonatology Unit of the Santa Chiara Hospital of Pisa (Italy). Inclusion criteria were term birth (gestational age of at least 37 weeks) an Apgar score, measured at 5 minutes after birth, of at least 7; and the parental subscription of an informed consent. Specifically, the Apgar score is a prognostic score used to assess the clinical conditions of newborns as soon after delivery. It is composed of 5 items (skin color, pulse, breathing, muscle tone, and reflex irritability) with a score for each one from 0 to 2. A normal Apgar score ranges from 7 to 10.

Exclusion criteria were defined as gestational age being lower than 37 weeks, an Apgar score lower than 7, suspicion of metabolic or genetic syndromes, or denied subscription of the parental informed consent. All subjects included in this study were of self-reported European ethnicity.

The Ethical Committee of Meyer Paediatric Hospital (Florence, Italy), which is the elected IRB for all paediatric studies in the Tuscany region of Italy, approved this study (registration number 84/2015). In addition, all clinical procedures were performed according to the ethical standards of the Declaration of Helsinki (1964).

### 2.2. Pain assessment

Following the guidelines defined by the Italian Neonatal Society for neonatal pain relief,^22^ all newborns were subjected to the oral administration of a 33% glucose solution a few minutes before the painful procedure, to provide nonpharmacological analgesia. The painful procedure consisted in a routine heel lancing for neonatal metabolic screenings.

The ABC scale, which has been validated in healthy term newborns,^6^ was used to assess pain after the administration of the therapy. The ABC scale is based on 3 parameters that evaluate crying intensity during the painful procedure: the pitch of the first cry, the constancy in time, and the rhythmicity of crying.^6^ A score from 0 to 2 is assigned to each parameter and then each value is added up, and therefore, the ABC score may vary from a minimum of 0 (no pain) to a maximum of 6 (maximum pain intensity).

All clinical procedures including pain assessment were performed by trained personnel of the Neonatology Unit of the Santa Chiara Hospital.

### 2.3. Variables under study

The environmental exposome was defined as the set of clinical and anthropometric variables potentially influencing the response to the analgesic treatment. More specifically, it comprised gestational age (weeks), maternal age (years), birthweight (kgs, as continuous variable), maternal pregravidic weight (kgs, as continuous variable), neonatal type of feeding (breastfeeding/partial breastfeeding/formula milk), maternal gestational diabetes (yes or no), pregravidic diabetes (yes or no), insulin assumption (yes or no), smoking status (yes or no), sex (male or female), delivery mode (vaginal or caesarean delivery), type of analgesia during delivery (spinal or epidural or total analgesia), and maternal analgesic drugs assumption during the last 3 months of pregnancy (yes or no). Particularly, the variables gestational diabetes and pregravidic diabetes refer to 2 different conditions. The former is a transient glucose metabolism impairment related to pregnancy, whereas the latter indicates that the mother had diabetes (usually type I, insulin dependent) before pregnancy. As some mothers with gestational diabetes and all those with pregravidic diabetes may be treated with insulin, the variable insulin assumption was included.

### 2.4. Single nucleotide polymorphisms selection

Single nucleotide polymorphisms (SNPs) within the *SLC2A1*, *SLC2A2*, and *SLC6A2* gene regions were selected based on a tagging approach to cover most of the genetic variability of these genes, using the Haploview Tagger Program software (https://www.broadinstitute.org/haploview/haploview, version 4.2).^5^ The criteria for the selection of tagging SNPs were minor allele frequency (MAF) > 0.05 and r^2^ < 0.8.

A set of 14, 3, and 19 SNPs was selected for *SLC2A1*, *SLC2A2*, and *SLC6A2* genes, respectively. For each SNP, information related to the chromosome, position, gene, minor allele frequency (MAF) in the 1000 Genomes database for the TSI population, and functional annotation of the SNP (missense, synonymous, intronic, and noncoding) is reported in supplementary files (Table ST1, available at http://links.lww.com/PAIN/B918).

### 2.5. DNA extraction and genotyping

Genomic DNA was extracted from blood cord with the automated QIAcube Connect extractor using the QIAamp DNA Blood Mini Kit as recommended by the producer. DNA concentration was quantified using a NanoDrop Lite UV–Vis Spectrophotometer (Thermo Fisher Scientific).

Genotyping was performed on 384 well plates using the TaqMan Assay system (Thermo Fisher Scientific, Waltham, MA), including 3.2% of duplicated samples for quality control purposes.

### 2.6. Statistical analysis

Hardy–Weinberg equilibrium was evaluated using the Pearson chi-square test.

Three different regression models were used to investigate the association between clinical and anthropometric variables and the response to the analgesic therapy. Logistic regression was applied to compare newborns with an ABC score > 0 with those with an ABC score = 0; a second logistic regression model was applied on a subset of individuals, to compare newborns not responding to the therapy and showing an high intensity of pain (ABC score ≥ 5) with those responding to the therapy (ABC score = 0). Finally, an ordered logistic regression model was performed comparing all newborns within each ABC score category “k” (with k assuming values from 0 to 5), with those within the next category “k + 1”, undertaking the proportional odds assumption.

The variables of the environmental exposome associated with the outcome at a *P*-value < 0.05 were selected and additionally evaluated according to Akaike information criterion (AIC) and Bayesian information criterion (BIC), for covariates selection. In brief, AIC and BIC are mathematical estimators of the goodness of fit of a regression model. They allow the identification of the optimal model among several ones differing only for the number of independent variables included, thus allowing the selection of the ideal set of covariates.

The analyses of association between SNPs and ABC score were performed under dominant and additive allelic inheritance models adjusted for the covariates identified in the previous step. The dominant model compares homozygous carriers for the less common allele and heterozygous subjects (grouped together) with the homozygous carriers for the more common allele. The assumption underlying this model is that one copy of the effect allele is enough to increase the risk for the outcome compared with the group of homozygous subjects for the noneffect allele and that the effect of one copy or 2 copies of the effect allele have the same effect on the phenotype. The additive allelic model, instead, assumes that homozygous individuals for the effect allele and heterozygous individuals have a 2-fold and 1-fold increased risk, respectively, of getting the phenotype of interest compared with homozygous subjects for the other allele.

Bonferroni correction was applied to define a study-wide threshold for statistical significance, by dividing 0.05 by the number of the independent variables tested: 0.05/(36 + 16) = 9.62 × 10^−4^. All statistical analyses were performed in RStudio, version 4.1.2.

### 2.7. Functional characterization of the single nucleotide polymorphisms

Several tools were used to investigate the functional effect of the SNPs associated with the response to the therapy. More specifically, the GTEx portal^4^ was used for identifying potential expression quantitative trait loci (eQTLs), which are SNPs that influence the expression of nearby genes; HaploReg v4.1^44^ and RegulomeDB 2.0.3^8^ were used to investigate the potential influence of the SNPs on DNA regulatory elements. Specifically, HaploReg provides data in relation to the effect of the SNPs on chromatin state and regulatory motifs. RegulomeDB provides a predictive score, intended as a probability score for the SNP to be functionally active, and a rank based on the amount of experimental evidence for the SNP to be functionally active. The rank goes from 1 to 6, where 1 indicates the highest evidence for functional or regulatory potential and 6 the lowest. The Combined Annotation Dependent Depletion (CADD) score was used to evaluate the deleteriousness of the SNPs.^35^

## 3. Results

### 3.1. Genotyping results and quality control

The average genotyping call rate for the 36 SNPs was 98.77%, and the concordance rate between duplicates was higher than 99%. The observed MAF, call rate, and genotypes distribution for each SNP are also reported in Table ST1 (http://links.lww.com/PAIN/B918).

*SLC2A1*-rs11537641 was not in Hardy–Weinberg equilibrium (*P*-value = 1.96 × 10^−15^), but since the distribution of the genotypes was very similar to that reported in 1000 genomes for the TSI population, it was not excluded from the following analysis.

### 3.2. Characteristics of the study population

The average gestational age was 39.59 ± 1.14 weeks, and the male–female ratio of the newborns was about 1:1 (Table 1). The nonpharmacologic therapy was highly effective, as 141 newborns (of 1421) did not respond to the treatment. Among them, 8 had an ABC score of 1, 59 of 2, 38 of 3, 1 of 4, 21 of 5, and 14 of 6. For a subset of 54 subjects, the ABC score information was not available. Complete information on the variables of the exposome is reported in Table 1.

**Table 1:** Characteristics of the study population.

Variable	ABC score = 0 (n = 1196)		ABC score > 0 (n = 141)	
	N*	Average ± SD†	N*	Average ± SD†
Gestational age, wk	1194/1196	39.58 ± 1.13	141/141	39.60 ± 1.12
Maternal age, y	1195/1196	33.89 ± 5.31	141/141	34.72 ± 5.01
Birthweight, kg	1192/1196	3.33 ± 0.43	141/141	3.30 ± 0.40
Maternal pregravidic weight, kg	1184/1196	62.91 ± 12.67	141/141	62.30 ± 11.15
Feeding type	Breastfeeding: 803 Partial breastfeeding: 331 Formula milk: 54	—	Breastfeeding: 91 Partial breastfeeding: 44 Formula milk: 5	—
Maternal gestational diabetes	Yes: 197 No: 992	—	Yes: 28 No: 113	—
Maternal pregravidic diabetes	Yes: 11 No: 1178	—	Yes: 3 No: 137	—
Maternal insulin assumption	Yes: 62 No: 1134	—	Yes: 13 No: 128	—
Maternal smoking status	Yes: 56 No: 1130	—	Yes: 4 No: 132	—
Delivery mode	Vaginal delivery: 667 Caesarean section delivery: 529	—	Vaginal delivery: 79 Caesarean section delivery: 62	—
Sex	Male: 629 Female: 567	—	Male: 65 Female:76	—
Spinal anaesthesia	Yes: 505 No: 679	—	Yes: 56 No: 83	—
Epidural anaesthesia	Yes: 241 No: 944	—	Yes: 30 No: 109	—
Total anaesthesia	Yes: 11 No: 1077	—	Yes: 1 No: 119	—
Analgesic drugs assumption	Yes: 94 No: 1085	—	Yes: 13 No: 121	—

The table reports the number of subjects for each variable based on the ABC score group (ABC score = 0 or ABC score > 0). In addition, the average and standard deviation values are reported for numeric variables, whereas the number of subjects for each level is additionally specified for categorical variables.

* It indicates the number of subjects for each variable.

† It stands for standard deviation.

### 3.3. Association between the exposome and response to the therapy

None of the tested variables was associated with the response to the analgesic treatment (*P* < 0.05) in the model comparing newborns responding to the therapy with newborns not responding to the therapy (Table 2). However, a suggestive association was observed between maternal insulin assumption and a lower analgesic efficacy, with an OR of 1.86 (95% CI 0.96-3.37) and a *P*-value = 0.052.

**Table 2:** Association between clinical and anthropometric variables and response to the therapy.

Variable	Logistic regression 1*		Logistic regression 2†		Ordered logistic regression‡	
	OR (95% CI)	*P*	OR (95% CI)	*P*	OR (95% CI)	*P*
Sex (female vs male)	0.77 (0.54-1.09)	0.145	0.71 (0.35-1.40)	0.321	0.88 (0.62-1.25)	0.475
Gestational age, wk	1.02 (0.87-1.19)	0.848	0.83 (0.61-1.13)	0.243	**0.82 (0.70-0.96)**	**0.013**
Maternal age, y	1.03 (1.00-1.07)	0.081	1.00 (0.94-1.07)	0.988	**1.05 (1.01-1.08)**	**0.011**
Birthweight, g	1.00 (1.00-1.00)	0.890	1.00 (1.00-1.00)	0.821	1.00 (1.00-1.00)	1.000
Pregravidic weight, kg	1.00 (0.98-1.01)	0.590	1.00 (0.97-1.03)	0.980	1.01 (0.99-1.02)	0.393
Feeding (partial breastfeeding vs breastfeeding)	1.17 (0.80-1.71)	0.413	**0.33 (0.10-0.86)**	**0.041**	1.15 (0.78-1.67)	0.483
Feeding (formula milk vs breastfeeding)	0.82 (0.28-1.91)	0.674	1.17 (0.19-4.07)	0.830	0.81 (0.28-1.89)	0.656
Maternal gestational diabetic status (negative vs positive)	1.25 (0.79-1.91)	0.325	0.96 (0.32-2.32)	0.928	1.25 (0.80-1.98)	0.330
Maternal pregravidic diabetic status (negative vs positive)	2.35 (0.53-7.62)	0.195	—	—	2.47 (0.69-8.86)	0.167
Maternal insulin assumption (no vs yes)	1.86 (0.96-3.37)	0.052	1.15 (0.18-3.90)	0.853	1.43 (0.72-2.85)	0.312
Maternal smoking habit (negative vs positive)	0.61 (0.18-1.52)	0.349	1.44 (0.23-4.95)	0.623	1.62 (0.78-3.37)	0.195
Delivery mode (vaginal delivery vs caesarean delivery)	0.99 (0.69-1.41)	0.953	1.31 (0.66-2.60)	0.444	1.11 (0.78-1.59)	0.550
Spinal anaesthesia (no vs yes)	0.91 (0.63-1.29)	0.594	1.08 (0.52-2.19)	0.828	1.09 (0.75-1.56)	0.659
Epidural anaesthesia (no vs yes)	1.08 (0.69-1.63)	0.731	0.69 (0.23-1.67)	0.456	0.68 (0.41-1.11)	0.121
Total anaesthesia (no vs yes)	0.82 (0.05-4.29)	0.852	—	—	—	—
Analgesics assumption (no vs yes)	1.24 (0.65-2.21)	0.489	1.90 (0.64-4.62)	0.193	0.75 (0.36-1.58)	0.449

Table 2 reports the association results for the 3 regression models for each clinical and anthropometric variable of the environmental exposome. Associations with P-value < 0.05 are reported in bold.

* A logistic regression analysis was performed on all individuals to compare individuals not responding to the therapy with individuals responding to the therapy.

† A logistic regression analysis was applied on a subset of individuals, considering nonresponding subjects with a high ABC score (≥5) with responding newborns.

‡ An ordered logistic regression analysis was performed on all individuals, comparing subjects within a ABC score category with those within the higher-level ABC score category.

When comparing newborns responding (ABC score = 0) to the therapy with newborns with an ABC score ≥ 5, partial breastfeeding (ie, feeding modality comprising both maternal breastfeeding and formula feeding) increased 3 times the probability of responding to the therapy compared with complete breastfeeding, with an OR of 0.33 (95% CI 0.10-0.86) and *P*-value = 0.041.

Two associations were observed in the ordered logistic regression model. Gestational age was associated with a higher analgesic efficacy, with an OR of 0.82 (95% CI 0.70-0.96) and *P*-value = 0.013 for each 1 week increase in gestational age. Instead, maternal age was associated with a lower analgesic efficacy with an OR of 1.05 (95% CI 1.01-1.08) and *P*-value =0.011 for each 1 year increase in maternal age. All results are reported in Table 2.

However, none of the 3 variables (feeding type, gestational age, and maternal age) was included as covariate in the genetic models after evaluation of AIC and BIC criteria.

### 3.4. Association between genetic variants and response to the analgesic therapy

After multiple-testing correction, one statistically significant association was observed for *SLC2A1*-rs1105297. The carriers of the G allele had a fourfold decreased chance of responding to the therapy in the model comparing newborns with ABC score =0 and newborns with ABC score ≥ 5: ORs of 3.98 (95% CI 1.95-9.17, *P*-value=4.05 × 10^−4^) and 4.18 (95% CI 1.88-10.10, *P*-value = 7.17 × 10^−4^) in the additive allelic and in the dominant models, respectively (Table 3).

**Table 3:** Association between genetic variants and response to the therapy.

SNP	Gene	EA*	NEA†	Genetic model	Logistic regression 1‡		Logistic regression 2§		Ordered logistic regression‖	
					OR (95% CI)	*P*	OR (95% CI)	*P*	OR (95% CI)	*P*
rs1105297	*SLC2A1*	G	A	Allelic model	1.38 (1.03-1.87)	**0.036**	3.98 (1.95-9.17)	**4.05 × 10** ^**−**^ **^4^**	1.40 (1.04-1.89)	**0.026**
				Dominant model	1.47 (0.98-2.20)	0.063	4.18 (1.88-10.10)	**7.17 × 10** ^**−**^ **^4^**	1.51 (1.01-2.25)	**0.045**
rs11210769	*SLC2A1*	C	T	Allelic model	1.85 (1.10-3.32)	**0.028**	4.69 (1.30-33.33)	**0.050**	1.83 (1.06-3.13)	**0.029**
				Dominant model	1.82 (1.05-3.32)	**0.039**	4.85 (1.30-34.48)	**0.048**	1.81 (1.03-3.17)	**0.040**
rs11537641	*SLC2A1*	G	A	Allelic model	1.77 (1.15-2.78)	**0.012**	3.15 (1.26-9.62)	**0.024**	1.78 (1.15-2.78)	**0.009**
				Dominant model	1.76 (1.14-2.78)	**0.012**	3.15 (1.26-9.62)	**0.024**	1.78 (1.15-2.75)	**0.013**
rs12446977	*SLC6A2*	G	A	Allelic model	1.35 (1.01-1.80)	**0.041**	1.96 (1.16-3.33)	**0.012**	1.37 (1.03-1.82)	**0.031**
				Dominant model	1.58 (1.05-2.38)	**0.029**	2.76 (1.25-6.61)	**0.016**	1.60 (1.07-2.40)	**0.024**
rs3820546	*SLC2A1*	A	G	Allelic model	1.33 (1.00-1.77)	**0.047**	1.27 (0.75-2.17)	0.377	1.32 (1.00-1.75)	0.052
				Dominant model	1.67 (1.05-2.74)	**0.036**	1.93 (0.80-5.52)	0.174	1.69 (1.05-2.73)	**0.031**
rs3820548	*SLC2A1*	G	A	Allelic model	1.52 (1.10-2.13)	**0.013**	1.31 (0.73-2.46)	0.381	1.49 (1.09-2.08)	**0.015**
				Dominant model	1.53 (1.01-2.30)	**0.043**	1.32 (0.60-2.85)	0.484	1.50 (1.00-2.25)	**0.049**
rs13330300	*SLC6A2*	G	A	Allelic model	1.41 (0.96-2.15)	0.094	3.28 (1.31-11.11)	**0.025**	1.41 (0.93-2.08)	0.099
				Dominant model	1.42 (0.90-2.29)	0.143	3.42 (1.28-12.05)	**0.027**	1.42 (0.89-2.26)	0.143
rs40434	*SLC6A2*	G	A	Allelic model	1.30 (0.97-1.75)	0.075	1.77 (1.02-3.13)	**0.045**	1.32 (0.99-1.77)	0.06
				Dominant model	1.24 (0.81-1.91)	0.326	2.13 (0.91-5.67)	0.101	1.26 (0.83-1.92)	0.283
rs710216	*SLC2A1*	A	G	Allelic model	1.19 (0.84-1.69)	0.326	2.99 (1.23-7.27)	**0.016**	1.22 (0.87-1.73)	0.248
				Dominant model	1.28 (0.84-1.93)	0.249	3.06 (1.21-7.73)	**0.018**	1.31 (0.87-1.98)	0.192
rs710222	*SLC2A1*	A	G	Allelic model	1.02 (0.78-1.35)	0.873	1.66 (0.97-2.84)	0.065	1.01 (0.77-1.33)	0.922
				Dominant model	1.09 (0.71-1.68)	0.689	2.54 (1.19-5.42)	**0.016**	1.15 (0.57-1.34)	0.532
rs1800887	*SLC6A2*	C	T	Allelic model	1.20 (0.85-1.72)	0.323	2.18 (0.98-4.88)	0.057	1.19 (0.75-1.76)	0.322
				Dominant model	1.16 (0.56-1.31)	0.493	2.66 (1.12-7.41)	**0.039**	1.17 (0.56-1.30)	0.455

The table reports all associations below the threshold of 0.05 (bold text) in at least 1 of the 3 regression models.

* Effect allele (allele increasing the risk of not responding to the therapy).

† Noneffect allele (allele decreasing the risk of not responding to the therapy).

‡ The logistic regression analysis was performed on all individuals, comparing nonresponding newborns with newborns responding to the analgesic therapy.

§ The logistic regression analysis was performed on a subset of individuals, comparing those with a high ABC score with responding subjects.

‖ The ordered logistic regression analysis was performed on all subjects, comparing those within a specific ABC score category with those within the next ABC score category.

All other associations below the threshold of 0.05 in at least 1 of the 3 regression models are reported in Table 3. Notably, 3 SNPs were consistently associated with the ABC score throughout all regression models: *SLC2A1*-rs11210769, *SLC2A1*-rs11537641, and *SLC6A2*-rs12446977. However, none of them reached the adjusted threshold of statistical significance.

For all 3 SNPs, the largest ORs were observed in the model comparing newborns responding to the therapy with newborns with an ABC score ≥ 5. More specifically, the C allele of *SLC2A1*-rs11210769 was associated with a fivefold decreased probability of responding to the analgesic treatment, with ORs of 4.69 (95% CI 1.30-33.33; *P*-value = 0.050) and 4.85 (95% CI 1.30-34.48; *P*-value = 0.048), in the additive allelic and in the dominant models, respectively.

The G allele of *SLC2A1*-rs11537641 was associated with a threefold decreased probability of responding to the therapy, with an OR of 3.14 (95% CI 1.26-9.62) and a *P*-value = 0.024 in both the additive allelic and the dominant genetic models. For *SLC6A2*-rs12446977, instead, the carriers of the G allele had a 2 to almost threefold decreased chance of responding to the therapy, depending on the genetic model: ORs of 1.96 (95% CI 1.16-3.33; *P*-value = 0.012) and 2.76 (95% CI 1.25-6.61; *P*-value = 0.016), in the additive allelic and dominant models, respectively.

The results for all the 36 SNPs are reported separately for each regression model in supplementary material—Tables ST2-4, available at http://links.lww.com/PAIN/B918.

### 3.5. Functional characterization of the single nucleotide polymorphisms

*SLC2A1*-rs1105297 is an eQTL for the *SLC2A1-AS1* gene in the caudate and putamen of basal ganglia, with homozygous subjects for the G allele showing lower expression levels of *SLC2A1-AS1*. RegulomeDB assigned a rank of 5 to *SLC2A1*-rs1105297 (indicating a functional effect of the SNP in a transcription factor binding site or in a DNase site) and a score of 1.0 (corresponding to the maximum predicted value). In addition, both RegulomeDB and HaploReg suggested that the SNP could potentially modify transcription factor binding sites in several brain regions, such as the substantia nigra and the caudate. The CADD score was 0.261. No modification of gene expression was found for *SLC2A1*-rs11210769. However, HaploReg suggested an effect of the SNP on regulatory enhancer sequences in several brain regions, whereas RegulomeDB assigned a rank of 2b (indicating evidence for a functional effect in transcription binding sites) and a score of 0.8. Moreover, *SLC2A1*-rs11210769 is in perfect LD with 7 other intronic noncoding variants (namely, rs79580038, rs80184186, rs75009191, rs12038788, rs12407435, rs60023956, and rs112981157), all of which are characterized by similar functional profiles according to both HaploReg and RegulomeDB. The CADD score for this variant was 5.537. *SLC2A1*-rs11537641 was not reported to be an eQTL in brain regions or other tissues of relevance for this study. HaploReg suggested the SNP to modify the effect of an enhancer or a promoter sequence, whereas RegulomeDB assigned a rank of 4 and a score of 0.61. However, the CADD score was quite high, showing a value of 29.50.

For *SLC6A2*-rs12446977 instead, neither GTEx nor HaploReg supported a potential functional effect of the SNP. RegulomeDB, instead, assigned a rank of 5 and score of 0.59 and suggested an association with a low or quiescent chromatinic state of the DNA region surrounding the SNP in several brain structures, among which the substantia nigra and the caudate nucleus. In addition, *SLC6A2*-rs12446977 is in perfect LD with 4 other intronic variants (*SLC6A2*-rs4436775, *SLC6A2*-rs2397772, *SLC6A2*-rs12920735, and *SLC6A2*-rs1861647). According to both HaploReg and RegulomeDB, all 4 genetic variants affect the structure of several DNA regulatory motifs. The CADD score for this variant was 2.341. Table ST5 in supplementary material shows a summary of all the functional annotation of the 4 SNPs, available at http://links.lww.com/PAIN/B918.

## 4. Discussion

The oral administration of glucose-based solutions is an effective analgesic treatment to prevent neonatal pain^10^; however, around 10% of the newborns do not respond and still perceive pain. The underlying factors—especially genetic ones—contributing to the interindividual variability in response to the treatment have not been completely identified.

We investigated whether the exposome and the genetic variability of 3 transporters coding genes (namely, *SLC2A1*, *SLC2A2*, and *SLC6A2*) were associated with the differential response to the analgesic therapy in a population of more than 1400 healthy term newborns. This is the largest study up to date performed in this setting.

Despite analyzing a wide environmental exposome comprising many nongenetic variables, no statistically significant associations with the response to the therapy were identified. This may suggest that the exposome may not have a strong effect on the response to the therapy, or that we do not have enough statistical power to detect it, because of the relatively limited number of individuals with ABC score > 0. Considering Bonferroni correction for multiple testing, we identified the statistically significant association between the G allele of *SLC2A1*-rs1105297 and a lower analgesic efficacy. The G allele of this SNP decreases the expression of the *SLC2A1-AS1* gene according to GTEx, HaploReg, and RegulomeDB. *SLC2A1-AS1* is a long noncoding RNA that downregulates the expression of *SLC2A1*.^38^ The effect on the expression of *SLC2A1-AS1* is specific for the putamen and the caudate nucleus, which together constitute the dorsal striatum that is enriched for the expression of mu-opioid receptors.^26,48^ Several evidence suggest the role of the dorsal striatum in reward-related and motivation-related functions.^15,30,31^ For example, the stimulation of mu-opioid receptors in the dorsal striatum affects eating behavior and generates motivation to gain a reward.^15^ In addition, the putamen has been suggested to contribute to sensory aspects of pain.^40^

Therefore, as the glucose-induced analgesia is probably mediated by the release of endogenous opioids,^10,19,33^ the identification of a genetic variant specifically affecting the expression of *SLC2A1* in the dorsal striatum, implicated in opioids-mediated reward, supports the involvement of *SLC2A1* in this analgesic process.

In addition, we also observed that the C allele of *SLC2A1*-rs11210769 and the G allele of *SLC2A1*-rs11537641 were consistently associated with a lower analgesic efficacy throughout all regression models (although never reaching the Bonferroni-adjusted threshold).

Robust evidence for a functional effect in enhancer sequences in the brain, among which the caudate and the substantia nigra regions, were observed for *SLC2A1*-rs11210769 through HaploReg and RegulomeDB. The alteration of DNA binding sites for transcription factors or other regulatory proteins may directly affect the expression of *SLC2A1* or other genes in such brain regions, which are involved in reward-related functions.^15,30,31^In line with this hypothesis, HaploReg reported an alteration of a motif (id: M00984) recognized by the transcription factor phosphatidylethanolamine binding protein (PEBP), coded by *PEBP1*. A study on the expression of *PEBP1* in a mouse model highlighted a high expression level in the nucleus accumbens.^41^ As the involvement of the nucleus accumbens in reward, motivation and addiction are well established,^14,29^ it is likely that PEBP is involved in reward-related functions too.^24,41^ Thus, the modification of PEBP1 binding site (due to *SLC2A1*-rs11210769) in the nucleus accumbens may be an additional potential process regulating the response to the therapy.

*SLC2A1*-rs11537641 is instead a synonymous variant which may regulate the expression of *SLC2A1* by influencing the kinetics of mRNA translation, according to codon usage bias principle.^32^ In brief, codon usage bias refers to the concept for which gene expression is influenced by the differential usage of some synonymous codons over the others. As a consequence of this nonrandom usage, there is a varying bioavailability of different aminoacyl-tRNA carrying the same amino acids, which causes different synonymous codons to be translated at different rates, thus influencing the processing of a transcript.

HaploReg and RegulomeDB both suggest a moderate functional effect for *SLC2A1*-rs11537641 in enhancer and DNA regulatory sequences in several regions.

An increased expression of *SLC2A1* in the dorsal striatum may lead to a higher striatal glucose availability that in turn may affect the glucose-mediated reward and thus partially explain the differential response to the analgesic therapy.

An interesting association was also observed between the G allele of *SLC6A2*-rs12446977 and a lower analgesic efficacy of the therapy, which was consistent throughout all regression models. The involvement of *SLC6A2* in relation to opioids-mediated analgesia has been already investigated.^7,46^ For example, knock-out models for *SLC6A2* showed a higher effect of the endogenous opioids-related analgesia compared with wild-type animals because of an increase of extrasynaptic noradrenaline.^7^ However, no data have been published, to the best of our knowledge, on the effect of *SLC6A2*-rs12446977 (or other SNPs in LD with *SLC6A2*-rs12446977) on analgesia. Being an intronic variant, it may be possible that *SLC6A2*-rs12446977 is involved in the splicing process or that it is in LD with other functional SNPs. In line with this possibility, HaploReg reported the alteration of several DNA regulatory elements due to 4 intronic variants in perfect LD with *SLC6A2*-rs12446977 (*SLC6A2*-rs4436775, *SLC6A2*-rs2397772, *SLC6A2*-rs12920735, and *SLC6A2*-rs1861647). Such regulatory elements are recognized by different transcription factors and chromatin-remodeling proteins, which may influence the expression of *SLC6A2.*

A study focusing on the relationship between monoamine transporters and pain response after oral surgery identified a time-dependent association between *SLC6A2*-rs40434 and analgesia onset in adults.^20^ It is possible that a time-dependent relationship between the genetic variability of *SLC6A2* and analgesia may contribute to the reduced analgesic response observed for some newborns. However, since all newborns were subjected to heel prick puncture 5 minutes after the analgesic treatment, we are not able to explore this possibility. Anyway, as *SLC6A2*-rs40434 is not in LD with *SLC6A2*-rs12446977, our result represents an additional independent indication that the genetic variability of the *SLC6A2* locus contributes to the interindividual differences in the analgesic responses.

To the best of our knowledge, this is the largest pharmacogenetic study performed in newborns. Another strength is represented by the homogeneity of the neonatal population analyzed and the availability of several maternal and neonatal variables to account for a wide environmental exposome. Possible limitations of this study are the relatively small number of individuals that do not respond to the therapy, and the limited variability in several of the clinical variables analyzed. These 2 factors together limit our power to detect small effects (OR < 1.6) on the outcome for some of the variables tested (analgesics assumption, total and epidural anesthesia, insulin assumption, pregravidic diabetes, and smoking status). Therefore, it is likely that a larger sample size is required to gain sufficient statistical power to identify the genetic contribution of rare allelic variants and small effects of exposome variables.

In conclusions, we observed a study-wide significant association between the G allele of *SLC2A1*-rs1105297 and a lower analgesic efficacy of the therapy. As the G allele of this SNP also decreases the expression of the antisense RNA *SLC2A1-AS1* in the dorsal striatum, which is involved in reward-related functions, we hypothesized that the genetic variability of *SLC2A1* may affect the response to the therapy through the regulation of the striatal glucose availability. This result represents a significant step towards the accomplishment of personalized analgesic treatment, which in the future will allow assigning the best regimen according to the genetic variability of the patient, maximizing the chance of analgesic efficacy and minimizing the administration of low-effective treatments or related side effects.

## Conflict of interest statement

The authors have no conflicts of interest to declare.

## Appendix A. Supplemental digital content

Supplemental digital content associated with this article can be found online at http://links.lww.com/PAIN/B918.
