# Supplementary material for: The genetic variant SLC2A1-rs1105297 is associated with the differential analgesic response to a glucose-based treatment in newborns
Source: Pain. 2023 Sep 13;165(3):657–65. doi: 10.1097/j.pain.0000000000003051 (PMC10859852; doi:10.1097/j.pain.0000000000003051)
Supplement: Supplementary file 1 [file jop-165-657-s001.pdf]

Table ST1 – SNPs characteristics

Gene	SNP	Chromosome	Position[a]	MM[b]	Mm[c]	mm[d]	MAF[e]	MAF TSI[f]	Call Rate (%)[g]	Type of variant[h]
SLC2A1	rs11537641	1	42930743	GG (865)	GA (498)	AA (2)	0.18 (A)	0.17 (A)	98.13	Synonym variant
SLC2A1	rs3820546	1	42932414	AA (412)	AG (680)	GG (292)	0.48 (G)	0.47 (G)	99.50	Intronic variant
SLC2A1	rs3820548	1	42932725	GG (620)	GA (600)	AA (116)	0.31 (A)	0.26 (A)	96.05	Intronic variant
SLC2A1	rs3768029	1	42933316	CC (434)	CT (697)	TT (247)	0.43 (T)	0.48 (T)	99.07	Intronic variant
SLC2A1	rs841853	1	42935767	CC (565)	CA (646)	AA (163)	0.35 (A)	0.35 (A)	98.78	Intronic variant
SLC2A1	rs841852	1	42935828	CC (795)	CT (449)	TT (58)	0.22 (T)	0.19 (T)	93.60	Intronic variant
SLC2A1	rs841847	1	42937037	CC (697)	CT (576)	TT (113)	0.29 (T)	0.25 (T)	99.64	Intronic variant
SLC2A1	rs1105297	1	42945925	GG (559)	GA (624)	AA (197)	0.37 (A)	0.33 (A)	99.21	Intronic variant
SLC2A1	rs710222	1	42947982	AA (404)	AG (683)	GG (293)	0.46 (G)	0.41 (G)	99.21	Intronic variant
SLC2A1	rs7522674	1	42951110	CC (832)	CT (447)	TT (56)	0.21 (T)	0.17 (T)	95.97	Intronic variant
SLC2A1	rs7519412	1	42967799	GG (659)	GA (598)	AA (125)	0.31 (A)	0.26 (A)	99.35	Intronic variant
SLC2A1	rs841858	1	42933496	GG (915)	GT (418)	TT (43)	0.18 (T)	0.16 (T)	98.92	Intronic variant
SLC2A1	rs710216	1	42963086	AA (853)	AG (466)	GG (65)	0.22 (G)	0.20 (G)	99.50	Intronic variant
SLC2A1	rs11210769	1	42963672	CC (1083)	CT (255)	TT (13)	0.10 (T)	0.10 (T)	97.12	Intronic variant
SLC2A2	rs5398	3	170998041	GG (625)	GA (619)	AA (134)	0.32 (A)	0.31 (A)	99.07	Missense variant
SLC2A2	rs11924648	3	171000207	AA (966)	AG (382)	GG (42)	0.17 (G)	0.16 (G)	99.93	Intronic variant
SLC2A2	rs28720688	3	171011340	AA (1008)	AG (350)	GG (29)	0.15 (G)	0.15 (G)	99.71	Intronic variant
SLC6A2	rs747107	16	55661809	CC (943)	CT (389)	TT (41)	0.17 (T)	0.11 (T)	98.71	Intronic variant
SLC6A2	rs40519	16	55664156	GG (974)	GC (359)	CC (32)	0.15 (C)	0.17 (C)	98.13	Intronic variant
SLC6A2	rs40434	16	55665613	AA (512)	AG (667)	GG (204)	0.39 (G)	0.44 (G)	99.42	Intronic variant
SLC6A2	rs36026	16	55670883	CC (1055)	CA (297)	AA (23)	0.13 (A)	0.14 (A)	98.85	Intronic variant
SLC6A2	rs62028846	16	55678618	CC (984)	CT (370)	TT (33)	0.16 (T)	0.13 (T)	99.71	Intronic variant

Gene	SNP	Chromosome	Position[a]	MM[b]	Mm[c]	mm[d]	MAF[e]	MAF TSI[f]	Call Rate (%)[g]	Type of variant[h]
SLC6A2	rs1814269	16	55683116	GG (423)	GA (680)	AA (286)	0.45 (A)	0.46 (A)	99.86	Intronic variant
SLC6A2	rs13330300	16	55684686	GG (936)	GA (396)	AA (56)	0.18 (A)	0.14 (A)	99.78	Intronic variant
SLC6A2	rs9939709	16	55690921	GG (994)	GT (354)	TT (33)	0.15 (T)	0.12 (T)	99.28	Intronic variant
SLC6A2	rs1566652	16	55695212	GG (482)	GT (667)	TT (233)	0.41 (T)	0.43 (T)	99.35	Intronic variant
SLC6A2	rs1800887	16	55699677	TT (848)	TC (453)	CC (83)	0.22 (C)	0.17 (C)	99.50	Intronic variant
SLC6A2	rs6499773	16	55705094	TT (946)	TA (389)	AA (39)	0.17 (A)	0.13 (A)	98.78	3' UTR
SLC6A2	rs12923720	16	55658891	CC (1208)	CT (173)	TT (3)	0.07 (T)	0.07 (T)	99.50	Intronic variant
SLC6A2	rs1532701	16	55664115	AA (457)	AG (670)	GG (256)	0.43 (G)	0.38 (G)	99.42	Intronic variant
SLC6A2	rs28618083	16	55664751	TT (571)	TC (628)	CC (186)	0.36 (C)	0.30 (C)	99.57	Intronic variant
SLC6A2	rs187715	16	55670130	TT (1257)	TC (127)	CC (3)	0.05 (C)	0.06 (C)	99.71	Intronic variant
SLC6A2	rs16955591	16	55679874	CC (1026)	CT (340)	TT (22)	0.14 (T)	0.10 (T)	99.78	Intronic variant
SLC6A2	rs12446977	16	55684677	AA (650)	AG (578)	GG (156)	0.32 (G)	0.36 (G)	99.50	Intronic variant
SLC6A2	rs2279805	16	55695212	CC (374)	CT (664)	TT (325)	0.48 (T)	0.47 (T)	97.99	Intronic variant
SLC6A2	rs36007	16	55701626	CC (1162)	CT (165)	TT (9)	0.07 (T)	0.06 (T)	96.05	Intronic variant

[a] Position in base-pairs (GRCh38.p13); [b] homozygous subjects for the major allele; [c] heterozygous subjects; [d] homozygous subjects for the minor allele; [e] minor allele frequency calculated within our dataset; [f] minor allele frequency according to 1000 Genomes for the TSI population; [g] Genotyping call rate; [h] functional consequence of the variant within the gene region.

Table ST2 – Association between SNPs and response to the therapy (logistic regression 1)

SNP	EA	NEA	Allelic model		Dominant model	
			OR (95% CI)	p-value	OR (95% CI)	p-value
SLC2A1-rs1105297	G	A	1.38 (1.03-1.87)	0.036	1.47 (0.98-2.20)	0.063
SLC2A1-rs11210769	C	T	1.85 (1.10-3.32)	0.028	1.82 (1.05 - 3.32)	0.039
SLC2A1-rs11537641	G	A	1.77 (1.15-2.78)	0.012	1.76 (1.14-2.78)	0.012
SLC2A1-rs3768029	G	A	1.25 (0.94-1.68)	0.127	1.38 (0.88-2.20)	0.168
SLC2A1-rs3820546	G	A	1.33 (1.00-1.77)	0.047	1.67 (1.05-2.74)	0.036
SLC2A1-rs3820548	G	A	1.52 (1.10-2.13)	0.013	1.53 (1.01-2.30)	0.043
SLC2A1-rs710216	A	G	1.19 (0.78-1.35)	0.326	1.28 (0.84-1.93)	0.249
SLC2A1-rs710222	A	G	1.02 (0.78-1.35)	0.873	1.09 (0.71-1.68)	0.689
SLC2A1-rs7519412	T	G	0.91 (0.65-1.24)	0.545	0.84 (0.56-1.26)	0.393
SLC2A1-rs7522674	T	C	1.11 (0.77-1.57)	0.574	1.01 (0.66-1.54)	0.959
SLC2A1-rs841847	C	T	1.13 (0.81-1.55)	0.464	1.33 (0.88-2.00)	0.175
SLC2A1-rs841852	A	G	1.04 (0.72-1.49)	0.824	0.95 (0.62-1.46)	0.826
SLC2A1-rs841853	C	T	1.03 (0.75-1.40)	0.855	1.06 (0.71-1.62)	0.774
SLC2A1-rs841858	T	C	0.83 (0.56-1.20)	0.328	0.78 (0.50-1.19)	0.252
SLC2A2-rs11924648	C	T	1.01 (0.69-1.45)	0.960	1.09 (0.71-1.67)	0.685
SLC2A2-rs28720688	G	A	0.90 (0.57-1.38)	0.632	0.95 (0.59-1.50)	0.842
SLC2A2-rs5398	T	C	0.91 (0.66-1.25)	0.562	0.92 (0.61-1.39)	0.691
SLC6A2-rs12446977	A	C	1.35 (1.01-1.80)	0.041	1.58 (1.05-2.38)	0.029
SLC6A2-rs12923720	T	C	0.67 (0.33-1.23)	0.226	0.67 (0.33-1.26)	0.245
SLC6A2-rs13330300	G	A	1.41 (0.96-2.15)	0.094	1.42 (0.90-2.29)	0.143
SLC6A2-rs1532701	A	G	0.78 (0.58-1.04)	0.090	0.67 (0.44-1.02)	0.056
SLC6A2-rs1566652	G	A	1.09 (0.81-1.45)	0.571	1.26 (0.82-1.98)	0.299
SLC6A2-rs16955591	G	C	1.03 (0.67-1.54)	0.889	1.02 (0.64-1.58)	0.945
SLC6A2-rs1800887	A	G	1.20 (0.85-1.72)	0.323	1.16 (0.56-1.31)	0.493
SLC6A2-rs1814269	T	C	0.94 (0.71-1.24)	0.651	0.95 (0.62-1.48)	0.822
SLC6A2-rs187715	T	A	0.83 (0.40-1.54)	0.571	0.77 (0.35-1.52)	0.473
SLC6A2-rs2279805	G	A	1.16 (0.88-1.53)	0.296	1.54 (0.97-2.51)	0.072
SLC6A2-rs28618083	G	A	0.88 (0.65-1.17)	0.372	0.85 (0.57-1.28)	0.443
SLC6A2-rs36007	C	T	0.90 (0.48-1.58)	0.736	0.94 (0.49-1.70)	0.848

SNP	EA	NEA	Allelic model		Dominant model	
			OR (95% CI)	p-value	OR (95% CI)	p-value
SLC6A2-rs36026	A	G	1.11 (0.71-1.68)	0.644	1.00 (0.61-1.60)	0.997
SLC6A2-rs40434	T	C	1.30 (0.97-1.75)	0.075	1.24 (0.81-1.91)	0.326
SLC6A2-rs40519	T	C	1.00 (0.66-1.48)	0.997	0.93 (0.59-1.45)	0.753
SLC6A2-rs62028846	T	C	1.03 (0.69-1.52)	0.876	1.00 (0.64-1.53)	0.998
SLC6A2-rs6499773	A	C	0.86 (0.58-1.26)	0.461	0.84 (0.53-1.31)	0.450
SLC6A2-rs747107	T	G	0.84 (0.56-1.24)	0.400	0.84 (0.53-1.30)	0.439
SLC6A2-rs9939709	T	G	0.76 (0.49-1.16)	0.221	0.76 (0.46-1.22)	0.274

The table reports the results of the association analysis of the logistic regression model comparing non-responding newborns (ABC score > 0) with newborns responding to the therapy (ABC score = 0).

a – Effect allele (allele affecting the risk of not responding to the therapy compared to the non-effect allele); b – Non-effect allele (allele considered as baseline).

Table ST3 – Association between SNPs and response to the therapy (logistic regression 2)

SNP	EA[a]	NEA[b]	Allelic model		Dominant model	
			OR (95% CI)	p-value	OR (95% CI)	p-value
SLC2A1-rs1105297	G	A	3.98 (1.95-9.17)	4.05×10^{-4}	4.18 (1.88-10.10)	7.17×10^{-4}
SLC2A1-rs11210769	C	T	4.69 (1.30-33.33)	0.050	4.85 (1.30-34.48)	0.048
SLC2A1-rs11537641	G	A	3.15 (1.26-9.62)	0.024	3.15 (1.26-9.62)	0.024
SLC2A1-rs3768029	G	A	1.62 (0.95-2.82)	0.081	1.83 (0.77-5.10)	0.203
SLC2A1-rs3820546	G	A	1.27 (0.75-2.17)	0.377	1.93 (0.80-5.52)	0.174
SLC2A1-rs3820548	G	A	1.31 (0.73-2.46)	0.381	1.32 (0.60-2.85)	0.484
SLC2A1-rs710216	A	G	2.99 (1.23-7.27)	0.016	3.06 (1.21-7.73)	0.018
SLC2A1-rs710222	A	G	1.66 (0.97-2.84)	0.065	2.54 (1.19-5.42)	0.016
SLC2A1-rs7519412	T	G	0.75 (0.39-1.37)	0.371	0.70 (0.32-1.50)	0.361
SLC2A1-rs7522674	T	C	1.08 (0.54-2.06)	0.813	0.95 (0.42-2.04)	0.897
SLC2A1-rs841847	C	T	1.19 (0.63-2.18)	0.587	1.46 (0.68-3.18)	0.332
SLC2A1-rs841852	A	G	0.77 (0.35-1.54)	0.485	0.72 (0.29-1.63)	0.444
SLC2A1-rs841853	C	T	0.94 (0.50-1.72)	0.848	1.00 (0.46-2.22)	0.992
SLC2A1-rs841858	T	C	0.45 (0.17-1.02)	0.078	0.45 (0.16-1.08)	0.094
SLC2A2-rs11924648	C	T	1.19 (0.60-2.20)	0.600	1.36 (0.60-2.94)	0.443
SLC2A2-rs28720688	G	A	1.31 (0.59-2.66)	0.481	1.48 (0.64-3.28)	0.341
SLC2A2-rs5398	T	C	1.14 (0.64-1.99)	0.642	1.10 (0.52-2.37)	0.806
SLC6A2-rs12446977	A	C	1.96 (1.16-3.33)	0.012	2.76 (1.25-6.61)	0.016
SLC6A2-rs12923720	T	C	0.84 (0.24-2.24)	0.755	0.87 (0.24-2.40)	0.800
SLC6A2-rs13330300	G	A	3.28 (1.31-11.11)	0.025	3.42 (1.28-12.05)	0.027
SLC6A2-rs1532701	A	G	0.76 (0.44-1.31)	0.334	0.76 (0.36-1.68)	0.485

SNP	EA[a]	NEA[b]	Allelic model		Dominant model	
			OR (95% CI)	p-value	OR (95% CI)	p-value
SLC6A2-rs1566652	G	A	0.98 (0.56-1.69)	0.949	1.11 (0.51-2.60)	0.799
SLC6A2-rs16955591	G	C	0.54 (0.18-1.29)	0.210	0.55 (0.18-1.39)	0.240
SLC6A2-rs1800887	A	G	0.46 (0.19-0.95)	0.057	0.38 (0.14-0.90)	0.039
SLC6A2-rs1814269	T	C	0.86 (0.49-1.47)	0.577	0.81 (0.37-1.84)	0.595
SLC6A2-rs187715	T	A	0.28 (0.02-1.32)	0.216	0.28 (0.02-1.38)	0.215
SLC6A2-rs2279805	G	A	1.26 (0.75-2.12)	0.388	1.55 (0.67-4.06)	0.330
SLC6A2-rs28618083	G	A	0.80 (0.45-1.38)	0.438	0.80 (0.38-1.71)	0.564
SLC6A2-rs36007	C	T	0.70 (0.16-2.08)	0.569	0.70 (0.16-2.14)	0.582
SLC6A2-rs36026	A	G	0.71 (0.26-1.63)	0.457	0.58 (0.19-1.46)	0.281
SLC6A2-rs40434	T	C	1.77 (1.02-3.13)	0.045	2.13 (0.91-5.67)	0.101
SLC6A2-rs40519	T	C	0.44 (0.15-1.07)	0.098	0.44 (0.14-1.10)	0.106
SLC6A2-rs62028846	T	C	0.61 (0.23-1.38)	0.271	0.62 (0.23-1.46)	0.302
SLC6A2-rs6499773	A	C	0.48 (0.18-1.05)	0.097	0.39 (0.13-0.97)	0.062
SLC6A2-rs747107	T	G	1.17 (0.56-2.24)	0.662	1.22 (0.53-2.69)	0.627
SLC6A2-rs9939709	T	G	0.37 (0.11-0.92)	0.060	0.36 (0.10-0.98)	0.069

The table reports the results of the association analysis of the logistic regression model comparing non-responding newborns with a high ABC score (ABC score > 4) with newborns responding to the therapy (ABC score = 0).

a – Effect allele (allele affecting the risk of not responding to the therapy compared to the non-effect allele); b – Non-effect allele (allele considered as baseline).

Table ST4 – Association between SNPs and response to the therapy (ordered logistic regression)

SNP	EA[a]	NEA[b]	Allelic model		Dominant model	
			OR (95% CI)	p-value	OR (95% CI)	p-value
SLC2A1-rs1105297	G	A	1.40 (1.04-1.89)	0.026	1.51 (1.01-2.25)	0.045
SLC2A1-rs11210769	C	T	1.83 (1.06-3.13)	0.029	1.81 (1.03-3.17)	0.029
SLC2A1-rs11537641	G	A	1.78 (1.15-2.78)	0.009	1.78 (1.15-2.75)	0.013
SLC2A1-rs3768029	G	A	1.28 (0.96-1.71)	0.093	1.42 (0.90-2.23)	0.686
SLC2A1-rs3820546	G	A	1.32 (0.99-1.32)	0.052	1.69 (1.05-2.73)	0.024
SLC2A1-rs3820548	G	A	0.67 (0.48-0.92)	0.015	0.67 (0.44-1.00)	0.049
SLC2A1-rs710216	A	G	1.22 (0.87-1.73)	0.248	1.31 (0.87-1.98)	0.143
SLC2A1-rs710222	A	G	1.01 (0.77-1.33)	0.922	1.15 (0.57-1.34)	0.075
SLC2A1-rs7519412	T	G	0.90 (0.65-1.23)	0.507	0.84 (0.56-1.26)	0.376
SLC2A1-rs7522674	T	C	1.09 (0.77-1.55)	0.627	1.00 (0.66-1.53)	0.889
SLC2A1-rs841847	C	T	1.12 (0.82-1.55)	0.472	1.34 (0.89-2.01)	0.455
SLC2A1-rs841852	A	G	1.01 (0.71-1.45)	0.945	0.93 (0.61-1.43)	0.913
SLC2A1-rs841853	C	T	1.02 (0.75-1.39)	0.89	1.07 (0.71-1.62)	0.374
SLC2A1-rs841858	T	C	0.81 (0.56-1.18)	0.276	0.76 (0.50-1.70)	0.065
SLC2A2-rs11924648	C	T	1.01 (0.69-1.46)	0.973	1.09 (0.71-1.67)	0.473
SLC2A2-rs28720688	G	A	0.92 (0.59-1.42)	0.702	0.98 (0.62-1.55)	0.927
SLC2A2-rs5398	T	C	0.91 (0.66-1.26)	0.569	0.92 (0.62-1.38)	0.811
SLC6A2-rs12446977	A	C	1.37 (1.03-1.82)	0.031	1.60 (1.07-2.40)	0.906
SLC6A2-rs12923720	T	C	0.67 (0.35-1.29)	0.232	0.68 (0.35-1.32)	0.132
SLC6A2-rs13330300	G	A	1.41 (0.93-2.08)	0.099	1.42 (0.89 – 2.26)	0.031
SLC6A2-rs1532701	A	G	0.79 (0.60-1.05)	0.106	0.69 (0.46-1.04)	0.049

SNP	EA[a]	NEA[b]	Allelic model		Dominant model	
			OR (95% CI)	p-value	OR (95% CI)	p-value
SLC6A2-rs1566652	G	A	1.06 (0.80-1.41)	0.686	1.22 (0.79-1.88)	0.283
SLC6A2-rs16955591	G	C	0.99 (0.66-1.48)	0.948	0.97 (0.62-1.51)	0.647
SLC6A2-rs1800887	A	G	0.84 (0.59-1.19)	0.322	0.85 (0.56-1.30)	0.700
SLC6A2-rs1814269	T	C	0.92 (0.69-1.22)	0.558	0.91 (0.59-1.40)	0.887
SLC6A2-rs187715	T	A	0.77 (0.40-1.49)	0.446	0.72 (0.35-1.48)	0.425
SLC6A2-rs2279805	G	A	1.18 (0.89-1.55)	0.249	1.56 (0.97-2.49)	0.192
SLC6A2-rs28618083	G	A	0.88 (0.66-1.18)	0.392	0.86 (0.58-1.29)	0.532
SLC6A2-rs36007	C	T	0.89 (0.50-1.61)	0.706	0.93 (0.50-1.71)	0.552
SLC6A2-rs36026	A	G	1.06 (0.70-1.61)	0.784	0.97 (0.61-1.56)	0.396
SLC6A2-rs40434	T	C	1.32 (0.99-1.77)	0.060	1.26 (0.83-1.92)	0.987
SLC6A2-rs40519	T	C	0.96 (0.65-1.42)	0.838	0.90 (0.58-1.41)	0.156
SLC6A2-rs62028846	T	C	1.00 (0.68-1.48)	0.990	0.97 (0.63-1.49)	0.739
SLC6A2-rs6499773	A	C	0.86 (0.58-1.27)	0.446	0.84 (0.54-1.30)	0.734
SLC6A2-rs747107	T	G	0.87 (0.59-1.29)	0.492	0.88 (0.56-1.36)	0.214
SLC6A2-rs9939709	T	G	0.76 (0.50-1.17)	0.219	0.76 (0.47-1.24)	0.275

The table reports the results of the association analysis of the ordered logistic regression model comparing newborns within each ABC score category with those within the next category, under the proportional odds assumption.

a – Effect allele (allele affecting the risk of not responding to the therapy compared to the non-effect allele); b – Non-effect allele (allele considered as baseline).

Table ST5 – Functional characterization of the SNPs

SNP	Gene	GTEx		RegulomeDB			HaploReg	CADD
		**eQTL – Target gene[a]**	**eQTL – Tissue[b]**	**rank[c]**	**score[d]**	**Chromatine state[e]**	**Chromatine state[f]**	**CADD PHRED score**
rs1105297	*SLC2A1*	*SLC2A1-AS1*<br>*SLC2A1-AS1*	Caudate nucleus<br>Putamen	5	1	Enhancer activity	Enhancer activity	0.261
rs11210769	*SLC2A1*	-	-	2b	0.8	Enhancer activity	Enhancer activity	5.537
rs11537641	*SLC2A1*	-	-	4	0.61	Strong transcription	Enhancer activity	29.50
rs12446977	*SLC6A2*	-	-	5	0.59	Quiescent/Low activity	-	2.341

The table reports the functional characterization of the most relevant SNPs.

[a] – It indicates the gene whose expression is altered by the SNP in the first column.

[b] - It indicates the tissue in which the SNP (first column) affects the expression of the target gene (third column). For simplicity purposes, only the eQTL data in brain structures relevant for this study are reported.

[c] – The rank indicates a category based on experimental data supporting a functional activity of the SNP.

[d] – The score indicates a probability for the SNP to be a regulatory variant.

[e] - It indicates the chromatinic state basing on RegulomeDB data.

[f] - It indicates the chromatinic state according to HaploReg database.
